# Supplementary material for: An open-source framework for large-scale, flexible evaluation of biomedical text mining systems
Source: J Biomed Discov Collab. 2008 Jan 29;3:1. doi: 10.1186/1747-5333-3-1 (PMC2276192; doi:10.1186/1747-5333-3-1)
Supplement: Additional file 1 — Evaluation platform source code. The additional file contains the software evaluation framework discussed in this paper. The most current release can be downloaded from SourceForge.net [1]. See the accompanying README file for instructions on installation and use. Also included in the distribution is a collection of UIMA wrappers for some commonly used BioNLP tools and annotated corpora. [file 1747-5333-3-1-S1.zip › bionlp_uima_repository-1.0.1/docs/api/src-html/edu/uchsc/ccp/uima/util/UIMASyntacticAnnotation_Util.html]

```
001    package edu.uchsc.ccp.uima.util;
002    
003    /*
004     * 05/05/06
005     */
006    import java.util.ArrayList;
007    import java.util.Iterator;
008    import java.util.List;
009    
010    import org.apache.uima.cas.CASException;
011    import org.apache.uima.jcas.JCas;
012    import org.apache.uima.jcas.cas.FSArray;
013    
014    import edu.uchsc.ccp.uima.annotation.CCPTextAnnotation;
015    import edu.uchsc.ccp.uima.annotation.syntactic.CCPClauseAnnotation;
016    import edu.uchsc.ccp.uima.annotation.syntactic.CCPPhraseAnnotation;
017    import edu.uchsc.ccp.uima.annotation.syntactic.CCPSentenceAnnotation;
018    import edu.uchsc.ccp.uima.annotation.syntactic.CCPTokenAnnotation;
019    import edu.uchsc.ccp.uima.annotation.syntactic.CCPTypedDependency;
020    import edu.uchsc.ccp.uima.mention.CCPNonComplexSlotMention;
021    import edu.uchsc.ccp.uima.mention.CCPSlotMention;
022    import edu.uchsc.ccp.util.external.stanfordparser.InvalidTypedDependencyException;
023    import edu.uchsc.ccp.util.external.stanfordparser.TypedDependencyCls;
024    import edu.uchsc.ccp.util.external.stanfordparser.TypedDependencyCls_Util;
025    import edu.uchsc.ccp.util.nlp.mention.ClassMentionTypes;
026    import edu.uchsc.ccp.util.nlp.mention.SlotMentionTypes;
027    
028    /**
029     * This class has been constructed to automate the conversion of annotations with class mention types of "token", "phrase", "clause", "sentence" into
030     * TokenAnnotations, PhraseAnnotations, ClauseAnnotations, and SentenceAnnotations, respectively.
031     * 
032     * @author williamb
033     * 
034     */
035    public class UIMASyntacticAnnotation_Util {
036    
037        public static final boolean DEBUG = false;
038    
039        public static void normalizeSyntacticAnnotations(JCas jcas) {
040    
041            List<CCPTextAnnotation> annotationsToAdd = new ArrayList<CCPTextAnnotation>();
042            
043            /* get all text annotations in the CAS */
044            Iterator textAnnotIter = jcas.getJFSIndexRepository().getAnnotationIndex(CCPTextAnnotation.type).iterator();
045    
046            /*
047             * Initialize a list to store the annotations that will be removed that the end of this process. These annotation comprise "token" annotations
048             * that have been converted to TokenAnnotations, etc.
049             */
050            List<CCPTextAnnotation> syntacticAnnotationsToRemove = new ArrayList<CCPTextAnnotation>();
051    
052            /* cycle through all annotations in the cas, and add explicit TokenAnnotations, PhraseAnnotations, etc. when appropriate */
053            while (textAnnotIter.hasNext()) {
054                CCPTextAnnotation ccpTA = (CCPTextAnnotation) textAnnotIter.next();
055    
056                String mentionName = ccpTA.getClassMention().getMentionName();
057    
058                if (DEBUG) {
059                    System.out.println("DEBUG -- UIMASyntacticAnnotation_Util: " + ccpTA.getClassMention().getMentionName() + "; "
060                            + ccpTA.getBegin() + " -- " + ccpTA.getEnd() + ": " + ccpTA.getCoveredText());
061                }
062    
063                if (mentionName.equals(ClassMentionTypes.TOKEN)) {
064                    if (DEBUG) {
065                        System.out
066                                .println("DEBUG -- UIMASyntacticAnnotation_Util: Possible token annotation needing conversion to TokenAnnotation...");
067                    }
068                    /* if it's already a TokenAnnotation, then we don't have to do anything */
069                    if (!(ccpTA instanceof CCPTokenAnnotation)) {
070                        if (DEBUG) {
071                            System.out.println("DEBUG -- UIMASyntacticAnnotation_Util:... it was not a TokenAnnotation already.");
072                        }
073                        CCPTokenAnnotation tokenAnnotation = new CCPTokenAnnotation(jcas);
074                        try {
075                            UIMA_Util.swapAnnotationInfo(ccpTA, tokenAnnotation);
076                        } catch (CASException ce) {
077                            ce.printStackTrace();
078                        }
079    
080                        // /* set the Annotator */
081                        // tokenAnnotation.setAnnotator(ccpAnnotator);
082                        //
083                        // /* set the AnnotationSet(s) */
084                        // tokenAnnotation.setAnnotationSets(ccpAnnotationSets);
085    
086                        /* set POS */
087                        CCPNonComplexSlotMention ccpSlotMention = (CCPNonComplexSlotMention) UIMA_Util.getSlotMentionByName(ccpTA,
088                                SlotMentionTypes.TOKEN_PARTOFSPEECH);
089                        if (ccpSlotMention != null) {
090                            tokenAnnotation.setPartOfSpeech(UIMA_Util.getFirstSlotValue(ccpSlotMention));
091                        }
092    
093                        /* set token lemma */
094                        ccpSlotMention = (CCPNonComplexSlotMention) UIMA_Util.getSlotMentionByName(ccpTA, SlotMentionTypes.TOKEN_LEMMA);
095                        if (ccpSlotMention != null) {
096                            tokenAnnotation.setLemma((UIMA_Util.getFirstSlotValue(ccpSlotMention)));
097                        }
098    
099                        /* set token stem */
100                        ccpSlotMention = (CCPNonComplexSlotMention) UIMA_Util.getSlotMentionByName(ccpTA, SlotMentionTypes.TOKEN_STEM);
101                        if (ccpSlotMention != null) {
102                            tokenAnnotation.setStem((UIMA_Util.getFirstSlotValue(ccpSlotMention)));
103                        }
104    
105                        /* set token number */
106                        ccpSlotMention = (CCPNonComplexSlotMention) UIMA_Util.getSlotMentionByName(ccpTA, SlotMentionTypes.TOKEN_NUMBER);
107                        if (ccpSlotMention != null) {
108                            tokenAnnotation.setTokenNumber(Integer.parseInt(UIMA_Util.getFirstSlotValue(ccpSlotMention)));
109                        }
110    
111                        /* set typed dependencies */
112                        List<CCPSlotMention> tdSlotMentions = UIMA_Util.getMultipleSlotMentionsByName(ccpTA,
113                                SlotMentionTypes.TOKEN_TYPED_DEPENDENCY);
114    
115                        if (tdSlotMentions != null) {
116                            /* initialize a new FSArray to hold the CCPTypedDependencies */
117                            FSArray typedDependenciesArray = new FSArray(jcas, tdSlotMentions.size());
118                            for (int i = 0; i < tdSlotMentions.size(); i++) {
119                                CCPNonComplexSlotMention tdSM = (CCPNonComplexSlotMention) tdSlotMentions.get(i);
120                                String tdStr = UIMA_Util.getFirstSlotValue(tdSM);
121    
122                                TypedDependencyCls tdc;
123                                try {
124                                    tdc = TypedDependencyCls_Util.parseTypedDependencyClsString(tdStr);
125                                    CCPTypedDependency ccpTD = new CCPTypedDependency(jcas);
126                                    ccpTD.setDependentTokenNum(tdc.getDependentTokenNum());
127                                    ccpTD.setGovernorTokenNum(tdc.getGovernerTokenNum());
128                                    ccpTD.setRelation(tdc.getRelation());
129                                    ccpTD.addToIndexes();
130    
131                                    /* add it to the FSArray */
132                                    typedDependenciesArray.set(i, ccpTD);
133                                } catch (InvalidTypedDependencyException e) {
134                                    e.printStackTrace();
135                                }
136                            }
137                            /* now add the FSArray to the TokenAnnotation */
138                            tokenAnnotation.setTypedDependencies(typedDependenciesArray);
139                        }
140    
141                        /* Add the new TokenAnnotation to the CAS, and flag the old version for removal */
142                        syntacticAnnotationsToRemove.add(ccpTA);
143                        //tokenAnnotation.addToIndexes();
144                        annotationsToAdd.add(tokenAnnotation);
145                    }
146    
147                } else if (mentionName.equals(ClassMentionTypes.PHRASE)) {
148                    /* if it's already a PhraseAnnotation, then we don't have to do anything */
149                    if (!(ccpTA instanceof CCPPhraseAnnotation)) {
150                            CCPPhraseAnnotation phraseAnnotation = new CCPPhraseAnnotation(jcas);
151                        try {
152                            UIMA_Util.swapAnnotationInfo(ccpTA, phraseAnnotation);
153                        } catch (CASException ce) {
154                            ce.printStackTrace();
155                        }
156                        // /* set the Annotator */
157                        // phraseAnnotation.setAnnotator(ccpAnnotator);
158                        //
159                        // /* set the AnnotationSet(s) */
160                        // phraseAnnotation.setAnnotationSets(ccpAnnotationSets);
161    
162                        /* set the PhraseType */
163                        CCPNonComplexSlotMention ccpSlotMention = (CCPNonComplexSlotMention) UIMA_Util.getSlotMentionByName(ccpTA,
164                                SlotMentionTypes.PHRASE_TYPE);
165                        if (ccpSlotMention != null) {
166                            phraseAnnotation.setPhraseType(UIMA_Util.getFirstSlotValue(ccpSlotMention));
167                        }
168    
169                        /* Add the new PhraseAnnotation to the CAS, and flag the old version for removal */
170                        syntacticAnnotationsToRemove.add(ccpTA);
171    //                    phraseAnnotation.addToIndexes();
172                        annotationsToAdd.add(phraseAnnotation);
173                    }
174                } else if (mentionName.equals(ClassMentionTypes.CLAUSE)) {
175                    /* if it's already a ClauseAnnotation, then we don't have to do anything */
176                    if (!(ccpTA instanceof CCPClauseAnnotation)) {
177                            CCPClauseAnnotation clauseAnnotation = new CCPClauseAnnotation(jcas);
178                        try {
179                            UIMA_Util.swapAnnotationInfo(ccpTA, clauseAnnotation);
180                        } catch (CASException ce) {
181                            ce.printStackTrace();
182                        }
183    
184                        // /* set the Annotator */
185                        // clauseAnnotation.setAnnotator(ccpAnnotator);
186                        //
187                        // /* set the AnnotationSet(s) */
188                        // clauseAnnotation.setAnnotationSets(ccpAnnotationSets);
189    
190                        /* set the clauseType */
191                        CCPNonComplexSlotMention ccpSlotMention = (CCPNonComplexSlotMention) UIMA_Util.getSlotMentionByName(ccpTA,
192                                SlotMentionTypes.CLAUSE_TYPE);
193                        if (ccpSlotMention != null) {
194                            clauseAnnotation.setClauseType(UIMA_Util.getFirstSlotValue(ccpSlotMention));
195                        }
196    
197                        /* Add the new ClauseAnnotation to the CAS, and flag the old version for removal */
198                        syntacticAnnotationsToRemove.add(ccpTA);
199    //                    clauseAnnotation.addToIndexes();
200                        annotationsToAdd.add(clauseAnnotation);
201                    }
202                } else if (mentionName.equals(ClassMentionTypes.SENTENCE)) {
203                    /* if it's already a SentenceAnnotation, then we don't have to do anything */
204                    if (!(ccpTA instanceof CCPSentenceAnnotation)) {
205                            CCPSentenceAnnotation sentenceAnnotation = new CCPSentenceAnnotation(jcas);
206                        try {
207                            UIMA_Util.swapAnnotationInfo(ccpTA, sentenceAnnotation);
208                        } catch (CASException ce) {
209                            ce.printStackTrace();
210                        }
211    
212                        /* Add the new SentenceAnnotation to the CAS, and flag the old version for removal */
213                        syntacticAnnotationsToRemove.add(ccpTA);
214    //                    sentenceAnnotation.addToIndexes();
215                        annotationsToAdd.add(sentenceAnnotation);
216                    }
217    
218                } else {
219                    /* do nothing with anything other than token, phrase, clause, or sentence */
220                }
221    
222            }
223    
224            /* add the new annotations */
225            for (CCPTextAnnotation ccpTA: annotationsToAdd) {
226                ccpTA.addToIndexes();
227            }
228            
229            
230            /* Removed the now unnecessary SyntacticAnnotations */
231            for (CCPTextAnnotation ccpTA : syntacticAnnotationsToRemove) {
232                ccpTA.removeFromIndexes();
233            }
234    
235        }
236    
237    }
```
